# Treatment Approaches for MOG-Ab-Associated Demyelination in Children

**DOI:** 10.1007/s11940-019-0541-x

**Published:** 2019-01-22

**Authors:** Yael Hacohen, Brenda Banwell

**Affiliations:** 1 Department of Neuroinflammation, Queen Square MS Centre, UCL Institute of Neurology, London, UK; 2 Department of Paediatric Neurology, Great Ormond Street Hospital for Children, London, UK; 3 Department of Neurology and Department of Pediatrics, Children’s Hospital of Philadelphia, Perelman School of Medicine, University of Pennsylvania, Philadelphia, PA 19104 USA

**Keywords:** Acquired demyelination syndromes (ADS), Neuromyelitis optica spectrum disorder (NMOSD), Acute disseminating encephalomyelitis (ADEM), Myelin oligodendrocyte glycoprotein (MOG), Autoantibodies

## Abstract

**Purpose of review:**

The purpose of this review is to summarize current understanding regarding the treatment of myelin oligodendrocyte glycoprotein antibody (MOG-Ab)-associated demyelination in children. Emphasis is placed on the unique obstacles we face when predicting the risk of relapse and the important implications of such challenges when planning treatment protocols.

**Recent findings:**

MOG-Abs are consistently identified in a range of acquired demyelinating syndromes (ADS) in adults and children with a clinical phenotype distinct of MS and AQP4-Ab neuromyelitis optica spectrum disorder. Although initially thought to be associated with a benign disease, recent reports of children who are treatment-resistant and developed progressive disability over time raise important questions about how children with relapsing MOG-Ab disease should be managed.

**Summary:**

MOG-Abs are common in children with ADS with both monophasic and relapsing disease courses. Treatment of patients with MOG-Ab-associated demyelination includes management of acute relapses and chronic immunotherapy for those with relapsing disease. Emerging consensus supports distinction of treatment strategies from those typically used for relapsing remitting MS, and several groups debate whether to follow treatment protocols akin to those for AQP4-Ab NMOSD. A key challenge remains predicting the severity of the disease at onset. Collaborative international consensus to derive shared clinical evaluative platforms standardized biological and neuroimaging protocols which can be used clinically, and partnered research programs are required to advance personalized treatment for children with MOG-Ab-associated demyelination.

## Introduction

The myelin protein, myelin oligodendrocyte glycoprotein (MOG), is exclusively expressed in the central nervous system (CNS). Although MOG represents only a minor component (0.05%) of the myelin sheath, its location on the outermost lamellae [1] and on the cell surface of oligodendrocytes makes it highly immunogenic and available for antibody binding [2]. In animal models, antibodies identified following MOG induction appear to mediate or contribute directly to demyelination [3].

Antibodies to MOG (MOG-Abs) have been detected in 30–50% [2, 4] of children at first presentation of acquired demyelinating syndrome (ADS) [5], with two studies from the UK/France [4] and the Netherlands [6] suggesting that MOG-Abs identified at onset are associated with a non-MS disease course. The presence of MOG-Ab identifies a subset of adults [7–9] and children [10, 11] meeting the clinical and imaging criteria for neuromyelitis optica spectrum disorder (NMOSD) without antibodies to aquaporin-4. Identification of MOG-Ab at the time of incident ADS, however, does not predict risk of relapse, as the majority of MOG-Ab-positive children with acute disseminated encephalomyelitis (ADEM) or isolated optic neuritis (with normal brain MRI) experience a monophasic disease course.

Published cohort studies of adults [7–9] and children [10, 11] with NMOSD demonstrate that MOG-Abs associated with younger patients (particularly children) are frequently in males (and do not demonstrate the strong female preponderance seen in AQP4-Ab NMOSD), and despite the relapsing disease course, patients demonstrate good recovery from the acute relapses and the overall visual and motor outcome is better compared with AQP4-Ab-positive patients [12, 13].

A proportion of patients with MOG-Abs meet the McDonald 2017 criteria for a diagnosis of MS at onset and can experience relapses typical of MS posing significant diagnostic and treatment challenges. Features of such patients that render them “atypical” for MS will be presented.

Treatment of patients with MOG-positive demyelination includes management of acute relapses and chronic immunotherapy for those with relapsing MOG disease. Emerging consensus supports distinction of treatment strategies from those typically used for relapsing remitting MS, and several groups debate whether to follow treatment protocols akin to those for AQP4-Ab NMOSD [14, 15].

In this review, we will focus on pediatric-onset MOG-Ab-associated disease, with specific attention to challenging clinical scenarios. We will review data on the acute care of a demyelinating attack in addition to treatment of patient with relapsing diseases. Particular attention will be paid to the unique obstacles we face when predicting the risk of relapse and the important implications of such challenges when planning treatment protocols.

## Part 1: In clinical practice

### Presenting features

The clinical phenotypes of children with MOG-Ab-associated disease include monophasic ADEM, ADEM followed by recurrent optic neuritis (ON), or AQP4-negative NMOSD [11]. MOG-Abs are present in more than 30% of children who present with an initial episode of demyelination, in more than 50% of those presenting with ADEM, and in almost all those with multiphasic ADEM (MDEM) [16]. The prevalence of MOG-Ab stratified to the different demyelinating phenotypes is summarized in Table 1. Children with MOG-Ab are frequently Caucasian (in contrast to patients with AQP4-Ab who are more often non-Caucasians). In younger children, males are equally represented, with a slight female preponderance in adolescents (in contrast with the marked female preponderance seen in AQP4-Ab patients). Unlike AQP4-Ab-positive NMOSD, MOG-Ab does not link with other autoimmune diseases and was not reported in association with specific malignancy. Although many patients report prodromal illnesses, no specific viruses have been linked to MOG-related demyelination.

**Table 1:** A summary of key publications describing the frequency of MOG-Ab in children with ADS

	Hennes [17•]	Ketelsleger [6]	Fernandez-Carbonell [18]	Dale [19]	Fadda [20•]	Duignan [16]	Total
MOG-Ab (all ADS)	65/210 (30.9%)	21/1117 (17.9%)	13/74 (17.6%)	31/73 (42.4%)	99/279 (35.4%)	76/237 (32.1%)	305/990 (30.8%)
Relapsing disease in MOG-Ab+ children	25/65 (38.4%)	9/21 (42.8%)	8/13 (47.1%)	10/31 (32.2%)	18 (18.1%)	37/76 (49%)	107/305 (35.1%)
MOG-Ab positivity within phenotypes							
ADEM at onset	33/57 (57.9%)	16/24 (36.4%)	4/10 (40%)	11/24 (45.8%)	36/65 (55.4%)	45/70 (64.2%)	145/250 (58%)
ON at onset	12/24 (50%)	2/20 (10%)	8/28 (28.6%)	6/7 (85.7%)	29/85 (34.1%)	28/65 (43.1%)	85/229 (37.1%)
TM at onset	4/18 (22.2%)	0/7 (0%)	5/30 (16.7%)	4/13 (30.8%)	10/81 (12.3%)	3/50 (6%)	26/199 (13.1%)
MDEM/ADEM-ON	11/11 (100%)	5/5 (100%)	1 /2 (50%)	NA	NA	24/25 (96%)	41/43 (95.3%)
NMOSD	9/16 (56.3%)	3/3 (100%)	2/2 (100%)	NA	NA	13/33 (39.4%)	27/54 (50%)

MOG-Ab autoimmunity is not only more common in the young, but also appear to demonstrate age-dependent phenotypes, with brain involvement more frequently seen in younger children and ON and NMOSD in the older children (> 9 years) [21] and adults [22•].

Overall, in pediatrics, MOG-Abs are more common than AQP4-Ab. In children with ON, in whom about 50% will have MOG-Ab, involvement of the anterior optic pathway (papillitis) is more frequently seen as compared to AQP4-Ab-positive patients who more often have lesions involving the posterior pathway with both chiasmal and post chiasmal involvement [23]. In both MOG-Ab- and AQP4-Ab-related demyelination, bilateral ON and the presence of longitudinally extensive lesions extending down the optic pathway are distinguishing features, as these are atypical for isolated ON or for optic nerve relapses in MS.

Interestingly, MOG-Abs are uncommon in children presenting with isolated TM. In a recent report of 50 children with TM, only 3 (6%) were MOG-Ab-positive [16]. 39/50 (78%) who presented with TM were antibody-negative, and 36/50 (72%) experienced a monophasic disease course. The rare child who experiences relapsing TM typically either meets criteria for a diagnosis MS based on brain lesion pattern and non-spinal cord relapses or meets criteria for NMOSD (with antibodies to AQP4 or MOG). MOG-Ab-positive TM is almost always consistent with LETM, with central cord lesion involving the nerve roots and gray matter [24]. T2-bright lesions can extend all the way from the cervical spinal cord to the conus [25]. In contrast, in AQP4-Ab-positive patients spinal lesions are frequently in the cervico-thoracic spine and may extend superiorly to the brainstem (area postrema), while spinal lesions in MS are focal (< 3 spinal segments) and rarely traverse the full cross-sectional diameter of the cord [26]. When brain lesions are present identification of specific pattern may aid in the diagnosis, for example, when the brain lesions are typical for MS.

In general, MOG-Ab and AQP4-Ab patients differ in terms of clinical severity at onset, with a milder deficit in the MOG-Ab group [22, 27]. For example, MOG-Ab patients with T2 bright signal involving the entire spinal cord may have relatively mild weakness and sensory impairment and recover promptly and completely with acute treatment. This is in keeping with historical reports of children with ADEM (who were likely to have MOG-Ab) and myelopathy on MRI with no clinical signs to support the myelopathy. Despite the milder motor phenotype at onset, it is important to monitor for bowel and bladder problems at follow-up with a recent adult cohort reporting 28% bladder issues, 20% bowel dysfunction, and 21% of males had erectile dysfunction; all related to a previous transverse myelitis [27].

MOG-Abs are detected in over half the children presenting with ADEM and in nearly all patients who relapse following ADEM (MDEM, ADEM-ON, and NMOSD) [16, 17•, 20•]. No clinical features or brain MRI findings, at onset of ADEM, can distinguish between the MOG-Ab positive and ADEM without antibodies. Although initially thought to be associated with predominantly white matter disease, there are increasing reports of both adults [28, 29] and children [30••] with MOG-Ab-associated disease presenting with predominantly gray matter disease (some may have MRI findings restricted to the cortex) presenting with encephalopathy and seizures. Children presenting with a clinical phenotype of encephalopathy, seizures, and headaches associated with focal leptomeningeal enhancement have been diagnosed CNS vasculitis [30••]. Brain biopsies in some of these children support such a diagnosis, and many of the prior studies were reported prior to the ability to measure MOG-Ab [31].

## Part 2: Investigations

### Antibody detection assay

The different antibody detection methods are now an essential laboratory investigation in evaluating children with inflammatory CNS disorders, with diagnostic, prognostic, and therapeutic implications. Interpreting these assays requires the knowledge of the methodological strengths and weaknesses of each technique and is different for the different antigens identified (intracellular vs. extracellular). Earlier studies, which looked at antibodies to the linear epitopes of the denatured MOG protein using ELISA and Western blotting, resulted in inconsistent results and positivity in healthy controls [32]. Additionally, even when using the more specific cell-based assays, differences in positivity across cohorts were seen when the full-length protein construct is used, compared to the truncated form that lacks the intracellular domain. The reason for this difference is unknown, but recent studies suggest that the full-length protein can provide a specific and more sensitive assay for antibodies to MOG in patients with demyelinating disorders. Another technical obstacle with the MOG assay was the cross-reactivity of the IgG heavy and light secondary antibody with MOG-IgM and the need to use either specific IgG1 secondary antibody [33] or a secondary anti-Fc. To date, no comparative study to compare the sensitivity and specificity of these two methods has been performed, but both assays appear to identify patients with similar MOG-Ab phenotypes.

The relationship between MOG-Ab titers and clinical disease activity, however, remains an area of active investigation, with a recent report suggesting that a high MOG-Ab titer (≥ 1:1280) predicted a recurrent non-MS course with a sensitivity of 46% and a specificity of 86% [17•]. The presence of MOG-Ab over time, however, does not reliably predict relapses, as shown by a study demonstrating that persistence in antibody positivity in 35/43 (81%) of children did not differentiate between monophasic and relapsing patients (13/16 monophasic and 22/27 relapsing were persistently positive) [16]. Interestingly, two children with relapsing ADEM became seronegative in between attacks and were MOG-Ab-positive at the time of relapse. None of the children were MOG-Ab-negative at time of relapse. A large nationwide French study of 197 adults with MOG-Ab [22•], observed that the titters were higher at relapse than in remission, but only two patients (18.2%) became seronegative. Overall, the authors concluded that antibody titers were not reliable enough to use in the clinical setting for patient management.

### Neuroimaging

**Fig. 1:**
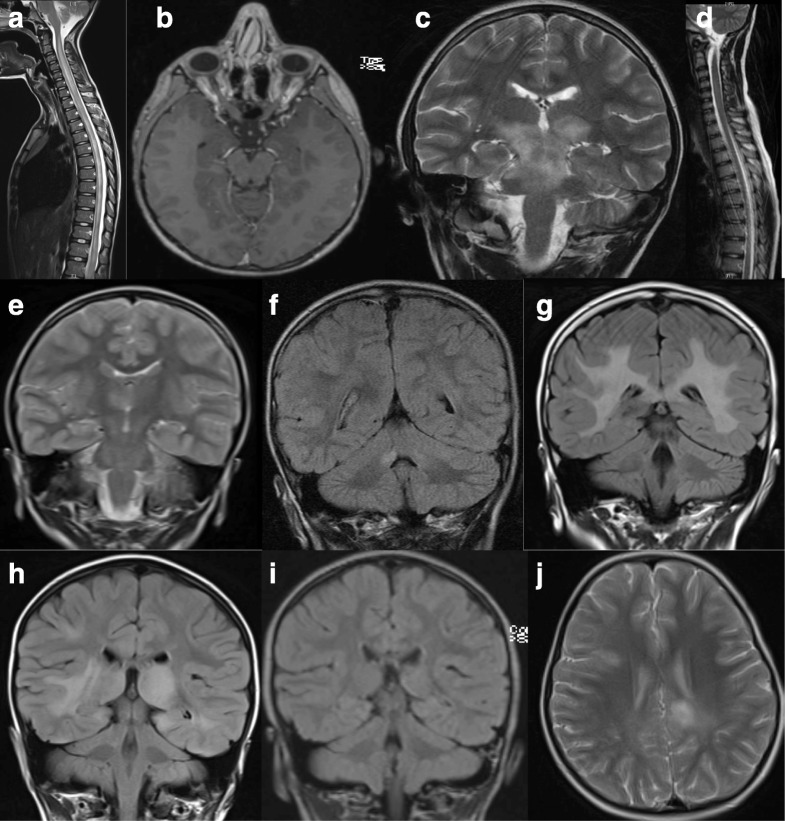
MRI findings in children with MOG-Ab-associated disease. A 10-year-old boy presented with encephalopathy, limb weakness (but able to walk), bilateral optic neuritis with only light perception, and urinary incontinence. MRI revealed longitudinally extensive transverse myelitis (**a**) and bilateral optic neuritis with enhancement (**b**). He relapsed 4 months later with headaches, fever, and encephalopathy. MRI revealed confluent lesions involving the pons, midbrain, and the superior cerebellar peduncles (**c**) as well as the left frontal opercular cortex. The previous spinal lesion has significantly improved (**d**). A 4-year-old girl presented with encephalopathy, seizures, and lethargy progressed to weakness of her right arm and bilateral 6th nerve palsies. MRI demonstrated a focus of signal change in the right thalamus (**e**). She relapsed 4 months later with brainstem cerebellar syndrome and lesions in both middle cerebellar peduncles (**f**) as well as a new lesion in the left side of the midbrain (not shown). At 4 years of age, she presented with bilateral optic neuritis. MRI showed diffuse bilateral white matter signal abnormality involving the cortical and subcortical regions extending inferiorly along the cerebral peduncles, pons, and right middle cerebellar peduncle, as well as swelling and signal abnormality of the corpus callosum (**g**). She had a further episode of bilateral optic neuritis 1 year later. A 4-year-old girl presented with encephalopathy, headache, and lethargy and MRI findings revealed diffuse bilateral and asymmetric signal abnormalities of the basal ganglia and thalamus with similar non-enhancing disease of the cortex, subcortical and deep white matter of both hemispheres (**h**). Repeat imaging 3 months later demonstrated near complete resolution of all lesions, concurrent with clinical recovery. She relapsed 15 months later with new onset headaches, encephalopathy characterized by behavioral change with hallucinations and confusion, and MRI evidence of new cortical and juxtacortical T2 lesions (**j**).

Neuroimaging studies in MOG-Ab patients should include the entire spine, optic nerves (if visual symptoms), and the brain. When present, T2 bright brain lesions typically involve white matter tracts, or can demonstrate large, hazy, ill-defined white matter lesions, and often involve deep gray matter (most commonly the thalamus) [24]. Imaging can usually be distinguished from MS by the absence of discrete, well-defined oval lesions in the periventricular white matter. Figure 1 illustrates some of the imaging patterns seen in children with MOG-Ab-associated disease. Children with MOG-Ab may present with four MRI patterns: (1) multifocal hazy/poorly marginated lesions, involving both gray matter and white matter and typically involving the middle cerebellar peduncles; (2) spinal cord and/or optic nerve involvement with normal intracranial appearance, or non-specific white matter lesions; (3) extensive and periventricular white matter lesions, resembling a “leukodystrophy-like” pattern; and (4) cortical encephalitis with leptomeningeal enhancement. Dramatic resolution, sometimes within a month of presentation, has been the radiological hallmark of MOG-Ab, perhaps suggesting that there is more edema than demyelination. Nevertheless, a proportion of children (predominantly very young patients) develop a “leukodystrophy-like” phenotype with large confluent lesions and significant brain atrophy mimicking a neurodegenerative disease [21]. Enhancement pattern can also aid in the diagnosis with ring/broken ring enhancement in MS [34], cloud-like enhancement in AQP4-Ab [35] and highly contrast enhancing lesion in the “leukodystrophy-like” phenotype [21]. This contrast enhancement may persist overtime even outside of a clinical attack.

### Optical coherence tomography

Optical coherence tomography (OCT) and electrodiagnostic tests can be useful paraclinical parameters in patients with ON [36•]. OCT quantifies retinal nerve fiber layer (RNFL) and ganglion cell layer thinning, and the development of microcystic macular edema and retinal damage. OCT offers an opportunity to monitor disease activity and progression non-invasively.

## Part 3: Clinical outcomes

The accumulation of disability in patients with antibody-mediated diseases, such as MOG-Ab, is thought to be very specifically relapse-related. Given the risk of disability due to incomplete relapse recovery, identifying patients at risk for relapse, and treating those with relapses, is the main focus of current management. Importantly, while MOG-Ab patients can experience a chronic disease with multiple relapses, some children experience a single event with a biphasic presentation (as defined by re-emergence of prior symptoms or expression of new symptoms within 90 days of initial presentation or at the time of weaning corticosteroids). Management of these two patient groups is clearly different.

Relapses following ADEM as defined by the International Pediatric Multiple Sclerosis Study Group (IPMSSG) revised criteria [37] may manifest as (i) recurrence of neurological symptoms within 3 months, often as immunomodulatory treatment is being weaned, considered to be a single protracted ADEM episode; (ii) second episode of ADEM after 3 months defined as “multiphasic” (MDEM) where there is either re-emergence of previous neurologic symptoms or new and different signs and magnetic resonance imaging (MRI) findings; or (iii) second clinical event is not associated with encephalopathy or occurs three or more months after the incident neurologic event [38].

Just as it is true in the clinical course, the radiological progression over time is different for the different syndromes with accrual of new focal lesions in MS (both symptomatic and clinically silent lesions) and symptomatic lesions only at time of clinical relapses in patients with AQP4-Ab NMOSD and MOG-Ab-associated demyelination, with significant resolution of the lesions in follow-up scans. Radiological evolution of myelitis (from short myelitis to LETM) over the first 12 days from presentation in a patient with AQP4-Ab NMOSD highlights that consideration of the timing of imaging is key and the time point to radiological nadir may be variable [39]. Radiological lag with worsening of the MRI during clinical recovery can be seen in children with MOG-Ab. This is also important to take into account at the time of follow-up scan; the first follow-up scan may not be suitable in identifying accrual of new lesions as the initial scan was only taken at one time point of a more dynamic process.

## Part 4: How to treat?

### Acute management

Currently, there are no evidence-based guidelines for the acute treatment of children with MOG-Ab. The current treatment approach typically focuses on removal of the systemic antibodies and immunosuppression. Of note, time from symptom onset to acute treatment in AQP4-Ab NMOSD is one of the major predictors of long-term outcome, as evidenced by a recent study of 29 adult patients with AQP4-Ab NMOSD, identifying an inverse correlation between the delay time pre-PLEX and EDSS score at 6-month post onset. Furthermore, the percentage reductions in EDSS score in groups receiving PLEX on days ≤ 15 and days 16 to 30 were significantly greater than those in the groups treated on days 31 to 60 and days 61 to 90 (all, *P* < 0.05) [40]. Although two large retrospective studies in adults [22•, 41] and one small retrospective study in children with MOG-Ab and ON [42] demonstrated favorable visual outcome, patients with MOG-Ab can manifest with severe visual loss and permanent paresis or ataxia [43]. A more aggressive, treatment-resistant, disease course may be seen in younger children in view of the increase susceptibility of the myelinating brain to MOG-Ab disease, as the autoantibody-mediated damage to the not fully matured (uncompact myelin) may result in secondary damage and irreversible axonal loss [44].

First-line immunotherapy normally consists of intravenous corticosteroids (30 mg/kg/day, maximum 1 g for 3–5 days), IVIG (total of 2 g/kg over 2 or 5 days), and plasma exchange (PLEX) in isolation or combination. PLEX with five to seven exchanges on alternative days is the best way of reducing antibody levels if urgently required, but not all centers will have the facilities. In pediatric, the tolerability of PLEX may also depend on the age and clinical presentation; an encephalopathic child with ADEM is less likely to tolerate plasma exchange than a teenager with transverse myelitis who is paralyzed. The sequence in which these therapies are given is also important as giving IVIG followed by plasma exchange will remove the IVIg from the systemic circulation. Steroids (intravenous methylprednisolone and/or high dose oral prednisolone) are thought to be very useful in reduction of inflammation, sealing of the blood brain barrier, and overall reduction of antibody production. Many of the patients with MOG-Ab, particularly the ones with abnormal brain MRI, have CSF leucocytosis and may initially be diagnosed as viral encephalitis [5, 30••]. Although corticosteroids were originally avoided in patients in whom the diagnosis of viral infection cannot be ruled out, evidence that corticosteroid administration with concurrent antibiotics/antiviral reduces neurological disability in immunocompetent children with various forms of CNS infections [45, 46] reduces concern regarding safety of corticosteroids acutely while the antibody results are pending.

The decision for how long to wean the corticosteroids for is dependent on the severity of the attack. With the clinical overlap between MOG and AQP4 antibody-associated diseases, many clinicians would not reduce the oral prednisolone below 20 mg per day (in the absence of maintenance immunosuppression) when the diagnosis of AQP4-Ab NMOSD is being considered. Therefore, as in most center the time to antibody results may take up to 3 weeks, many of the children (particularly the one presenting with optic neuritis or transverse myelitis) remain on steroids for over 4–6 weeks at the initial presentation. Chronic administration of corticosteroids has adverse effects on muscle, bone, mood, and the endocrine system and steroid-sparing medications might need to be considered if patients have repeated episodes when tapering of steroids is attempted. An association between post corticosteroids hypocortisolism with early first relapse was previously reported in children with idiopathic nephrotic syndrome with adrenocortical suppression being more common in younger children [47]. As the disease process in both nephrotic syndrome and MOG-Ab-associated disease is sensitive to glucocorticoid medication, differences in endogenous secretion of cortisol following prolonged treatment may be critical with younger children, perhaps more sensitive to the suppressive effect of prednisone when the dose is calculated per weight. This may result in iatrogenic relapses following weaning of medication.

### Maintenance treatment for relapse prevention

**Fig. 2:**
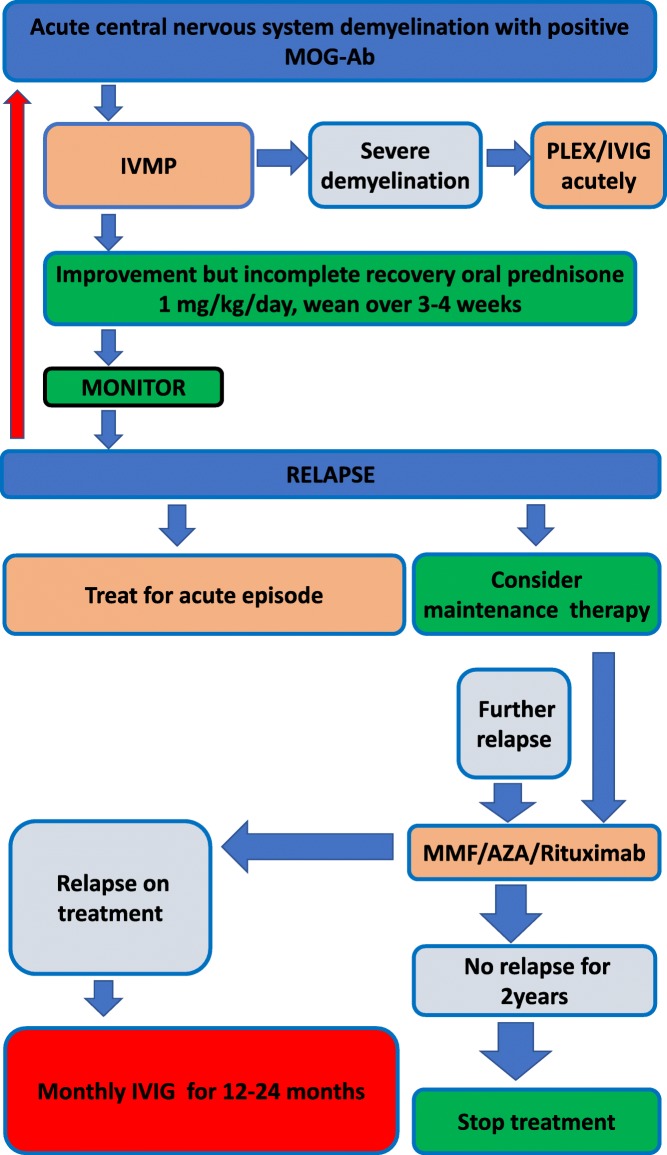
Treatment algorithm for children with MOG-Ab-associated disease. IVMP intravenous methylprednisolone, PLEX plasma exchange, IVIG intravenous immunoglobulin, AZA azathioprine, MMF mycophenalate mofetil.

Figure 2 illustrates a treatment algorithm for children with MOG-Ab. Following standard acute/induction therapy, a decision regarding the need for ongoing therapy for relapse prevention is typically influenced by (i) the likelihood of relapse or if more than one event time to relapse or annual relapse rate, (ii) the response to first-line treatment, and (iii) the severity of attack and the recovery from the initial attack. For example, if the child’s vision is poor in one eye, further relapse in the same eye or in the other eye may have a significant effect on the child disability and quality of life. In a study of 102 children with MOG-Ab-associated relapsing disease, only 52/102(51%) received maintenance treatment. The treated group had higher relapse rate and EDSS than the untreated group (median 1 relapse and EDSS 1.0 in the untreated group). This treatment paradox whereby the higher relapse rate and poorer outcome in the group having more therapy is simply reflected by the a-priori threshold for initiating such treatments. Nevertheless, these results suggest that at least a proportion of children may not require maintenance treatment.

Several case series advocate that typical first-line chronic MS treatment (interferon, glatiramer acetate) do not suppress relapses in MOG-Ab disease [30••, 48].

Despite the logic of using B cell depletion as a strategy for an antibody-associated disease, there are now increasing reports of patients with MOG-Ab who relapse on rituximab despite B cell depletion [30••]. In a study of 102 children with MOG-Ab-associated diseases, relapses were reported on all treatments (mycophenolate mofetil, azathioprine, rituximab, and cyclophosphamide) [30••]. The only treatment that appears to prevent relapses, particularly in children with multiple relapses a year, is repeated IVIG (every 4 weeks). IVIG is the only treatment that modifies the disease without leading to immunosuppression and risk of infection-triggered relapses. Interestingly, in a recent study using organotypic cerebellar slice cultures from transgenic mice and MOG-Ab-induced demyelination, IVIG was protective from demyelination in a dose-dependent manner. As binding to MOG antibody was not prevented, it was hypothesized to result from interference to complement-mediated oligodendroglial damage [49].

In cell culture, incubation with purified IgG from anti-MOG antibody-positive patients led to loss of the microtubule cytoskeleton of oligodendrocytes [19], and injection of purified IgG into the brains of mice resulted in myelin changes and altered expression of axonal proteins in the absence of inflammation, axonal loss, and neuronal or astrocyte death [50]. This evidence suggests MOG antibodies result in an autoimmune oligodendrogliopathy [51]. More generally, in antibody-mediated disorders, upregulation in the Th17 pathway, with raised CSF IL6, has been more commonly noted—a finding that is rare in MS [52].

One of the explanations for the heterogeneity in treatment response is that the MOG-Ab-specific damage is induced by multiple pathogenic mechanisms. A recent study looking at the effect of these antibodies in a T cell-mediated EAE animal models identified two pathogenic mechanisms both requiring specific T cell lines to cause the clinical disease [53]. (1) In synergy with MBP-specific T cells, patients derived MOG-Ab-induced active demyelination as seen in type 2 MS pathology (actively demyelinating lesions with loss of all myelin components, and immunoglobulin and complement deposition on the myelin) associated with profound blood brain barrier damage, activation of macrophages and local activation of terminal complement, an effect which was not seen with the T cells alone. (2) In synergy with cognate MOG-specific T cells, which by themselves do not induce clinical disease, the same affinity purified MOG-Ab preparation induced massively enhanced T cell infiltration and stimulation of microglia/macrophage infiltration in the subpial gray matter. It is not clear if these mechanisms affect all patients, occur at different stages of the disease or mediate different relapses, but these findings may suggest that targeting the antibody production alone may not be enough to stop the disease. Further studies on genetic susceptibilities, environmental triggers, and the role of the MHC complex in association with specific T cells may help in developing personalized medicine for children with MOG-Ab-associated disease.

## Part 5: Future directions

Meaningful advances in the diagnosis, prognostication, and treatment of MOG-Ab-positive patients requires a collaborative international consensus to derive shared clinical evaluative platforms, standardized biological and neuroimaging protocols which can be used clinically, and partnered research programs. Immunological signatures overtime may help when deciding on both treatment choices (targeting T cells vs B cells) and treatment duration. Consistent evaluation of patient outcomes is essential, but current outcome measures, such as the EDSS, used both clinically and for research, are not sufficiently sensitive.

A key challenge in selecting a suitable outcome measure. For younger MOG-Ab patients with an ADEM phenotype, cognitive evaluations are likely to be particularly informative. For MOG-Ab NMOSD, evaluation of visual function (including low contrast acuity, visual processing speed, and retinal nerve integrity) as well as gait assessment and evaluation of bladder function are more relevant. Inherent to the study of pediatric-onset disease, the full impact may not be immediately detected and may become more apparent as the child ages and fails to normally develop pathways that were injured during the acute phase of their illness.

The opportunity for early immunosuppressive treatment must be weighed against the potential to provide such therapy to a child destined for a monophasic illness for whom such prompt treatment is not required. At the present time, MOG-Ab titers, even when measured serially, do not reliably inform on such decisions. Future research will be invaluable in determining the immunological signatures that better identify children who require sustained immunosuppression. International collaboration will accelerate such discovery.
